# From Zero to Hero: Polymer Upcycling through Transformation of Waste PET Thermoforms into Kevlar

**DOI:** 10.1021/acsapm.5c00191

**Published:** 2025-04-24

**Authors:** Elanna P. Neppel, Richard-Joseph L. Peterson, Lars Peereboom, John R. Dorgan

**Affiliations:** Department of Chemical Engineering and Materials Science, Michigan State University, East Lansing, Michigan 48824, United States

**Keywords:** polymer upcycling, polymer recycling, circular economy, sustainable polymers, thermoforms, poly(ethylene terephthalate), terephthalamide, *p*-phenylenediamine

## Abstract

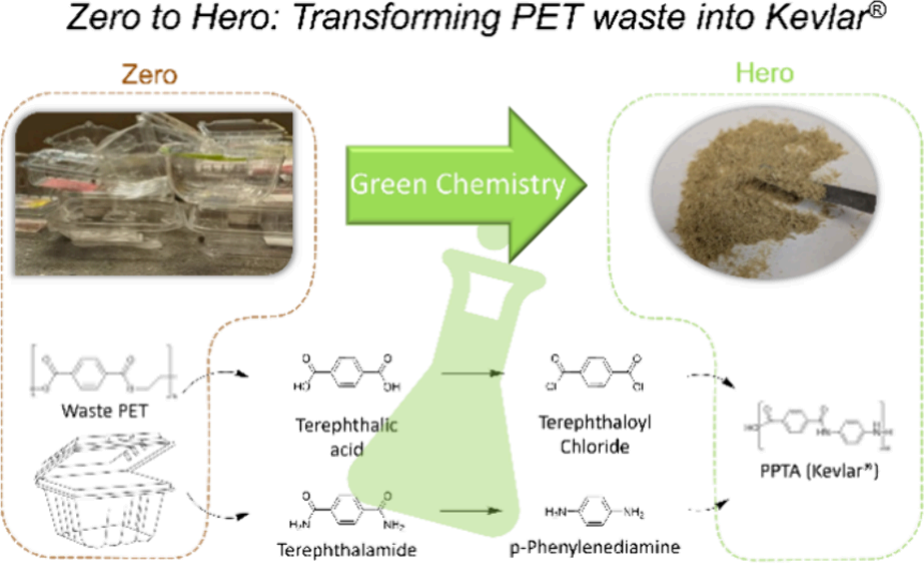

Polyethylene terephthalate (PET) is a widely used plastic packaging material that is often discarded after use. Previous studies have used recovered terephthalic acid derivatives to produce poly(*p*-phenyleneterephthalamide) (PPTA), an expensive commodity scale polymer widely known by the trade name Kevlar. Here, PPTA is synthesized using carbon that is 100% recovered from waste PET. To do so, the monomer *p*-phenylenediamine (PPD) is obtained through two facile “one-pot” reactions: (1) ammonolysis of PET to yield terephthalamide and (2) conversion of terephthalamide to PPD through a Hofmann type of rearrangement. Following earlier works, hydrolysis of PET followed by chlorination provides the monomer, terephthaloyl chloride (TCl). PPTA is synthesized by reacting the monomers in a solution of *n*-methyl pyrrolidone and calcium chloride. The pathway is demonstrated using zero-valued waste “clamshell” PET, a material usually excluded from recycling streams. The material reuse results in a lifesaving polymer used by members of the military, police, and other first-responders. It is concluded that this pathway provides an economic means of recovering and reusing waste PET that can reduce dependence on nonrenewable resources and foster greater material circularity in the plastics industries.

## Introduction

Plastics are produced on an enormous scale but are creating adverse effects on the environment and for human health. Uses for plastics span many applications such as fabrics, electronics, and single-use packaging.^1,2^ The historical demand for plastics has increased at an estimated annual growth rate of 8.4%; much of this increase is attributable to the rise of single-use plastics.^1,3^ Plastics that are improperly disposed of undergo weathering in the environment resulting in microplastic particles; such microplastic pollutants are globally distributed and found in human blood and organ systems.^1,3,4^ The growing demand for plastics, their sometimes deleterious environmental and health effects, and the drive for more efficient resource utilization, motivates the search for more sustainable practices at the very heart of Green Chemistry.^5,6^

Improved end-of-life scenarios can be envisioned for widely used polyethylene terephthalate (PET). The global use of PET creates 20 megatons of waste per year.^1,2,7^ Compared to other plastics, PET has a relatively high recovery rate, but economically viable PET recycling is centered around the recovery and reprocessing of bottles.^7,8^ However, PET is the polyester used in other applications, such as clothing, carpet,^9^ and in single use thermoformed containers (“clamshell” boxes and “blister” packaging).^10^ Containers made from PET are treated as contaminants in bottle recycling streams; there is an upper allowed limit, typically between 5 and 10% for thermoforms in PET bales.^8,10^ Due to these marketplace constraints, recycling methods specifically targeting PET thermoforms are needed.

Polymer upcycling uses chemistry to transform recovered plastics into more valuable materials.^11−14^ Chemical recycling refers to remaking the original source polymer. PET is chemically recycled at large scales using methanolysis and glycolysis.^11,12,15^ Significantly, the strategy proposed here for chemical upcycling (creating a more valuable polymer) is consistent with these commercial practices. because both methanol and glycol are suitable solvents for conducting ammonolysis. Furthermore, polyaramides, such as poly(*p*-phenyleneterephthalamide (PPTA, commonly known by its DuPont owned trademark Kevlar), are high value materials.^16−29^ Polyaramides sell for upward of $45 USD/kg (roughly 20x more valuable than PET!). In 2021, PPTA had a global market value of $449.2 which is projected to increase to $653.4 million by 2031.^27^ In fiber form, PPTA is often woven into fabrics; it is used in a wide variety of applications, including sports equipment, body armor, and structural composites.^17,20,22,23^

In a notable and innovative previous work, PPTA was synthesized from terephthalic acid (TPA) and terephthaloyl chloride (TCl) derived from waste PET.^30^ By using recovered TPA, the resulting PPD had a 55 wt % recycled content. On an industrial scale, this would translate to approximately 0.8 tons of PET diverted from a landfill per ton of PPTA produced. A number of other rigid-rod type of polymers were synthesized using other diamines and transamidization with diamines was also pursued. However, all diamines used, including the *p*-phenylenediamine (PPD), were purchased virgin materials derived from newly extracted fossil resources.

**Scheme 1:**
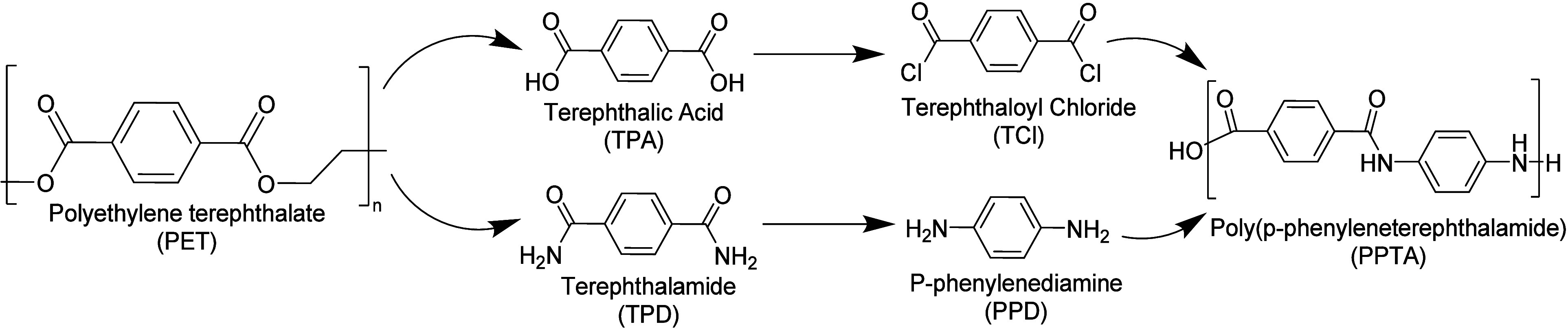
Synthesis Route from Waste PET to Much More Valuable Upcycled PPTA

**Figure 1:**
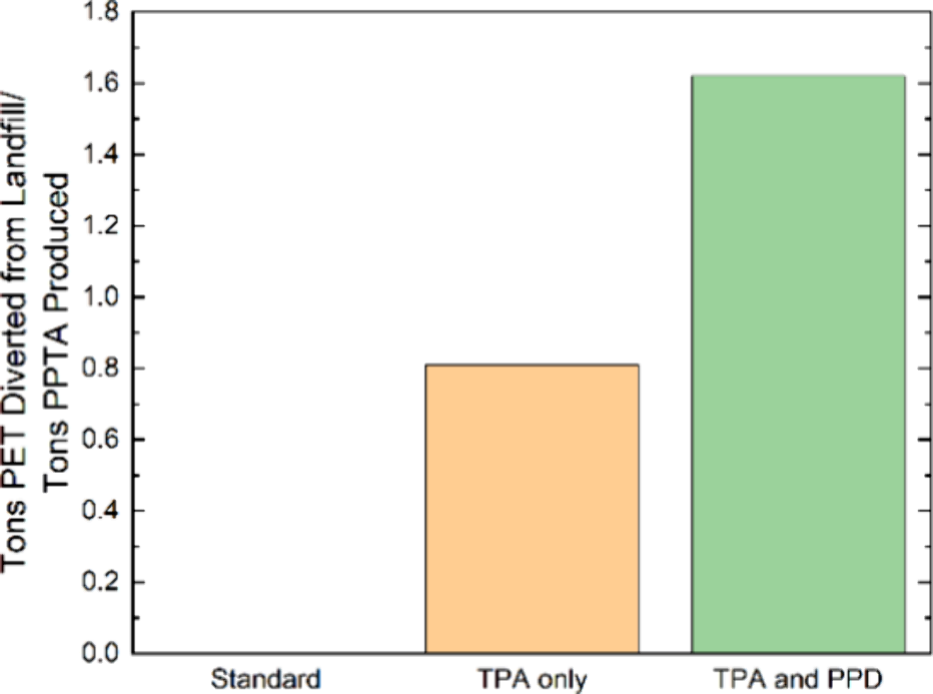
Recycled content for three different methods of PPTA production. There is no PET diverted in the standard, 0.81 tons PET/ton PPTA when TPA is obtained from waste PET, and 1.82 tons PET/ton PPTA when TPA and PPD are used.

In contrast, the PPD used here (Scheme 1) is also derived from waste PET and this offers significant economic and environmental advantages. The traditional route to PPD starts with the extremely energy intensive process of petroleum refining where crude oil is cracked, reformed, and separated to give a BTX (benzene-toluene-xylene) stream of aromatics.^31−33^ Additional energy intensive distillation is used to separate out benzene, a well-known carcinogen. PPD is then synthesized through a series of reactions starting with the chlorination of benzene which gives mono-, di-, and trisubstituted chlorobenzenes. An additional distillation is needed to isolate the 1-chlorobenzene. Next the chlorobenzene is preferentially nitrated (using strong acid) in the para-position. This disubstituted aromatic reacts with ammonia, evolving corrosive hydrochloric acid, and finally catalytically reduced using hydrogen (which has a large embedded energy content) to give the desired product. In the present alternative approach, waste PET is added to a saturated solution of ammonia in ethylene glycol and heated, the quantitative yield of diamide is treated with a mixture of bleach and caustic. With the addition of fresh caustic and bleach, the mixture can be recycled until sodium chloride precipitates as the only byproduct. Extraction is followed by a single distillation to obtain PPD. Because of the reduced number of distillations, energy can be saved. Also, the carcinogen benzene can be avoided by using waste PET as the source for making the PPD monomer. Furthermore, the recycled content increases from 55 wt % to 87% in the final polymer product. Figure 1 presents a comparison of the tons of landfill waste avoided per ton of PPD produced; additional quantification of the improved sustainability metrics are the subject of ongoing work.

## Materials and Methods

**Figure 2:**
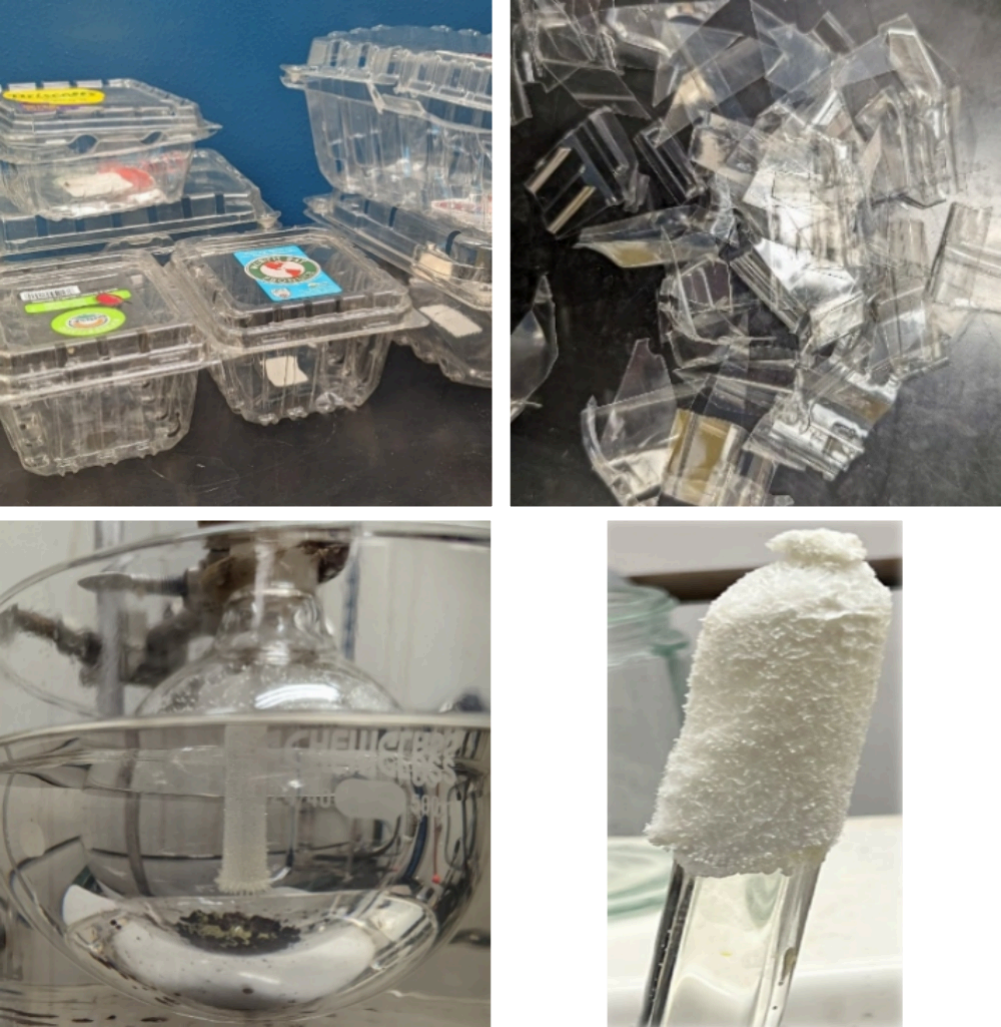
Material transformation. Waste PET thermoforms (top left) were washed with soap and water, dried, and cut into approximately 1 × 1 cm^2^ pieces before depolymerization (top right). Purified monomers are synthesized from the depolymerization, PPD is shown bottom left and TCl at bottom right.

PET waste was collected at the Michigan State University Recycling Center (see Figure 2). The collected waste was hand washed with dish soap and water. After washing, the material was rinsed, air-dried, and cut into square flakes with an edge length of approximately 1 cm; typical thickness was 0.40 mm.

Reagents were purchased from Sigma-Aldrich (St. Louis, MO) and prepared as described. Kevlar 29 woven fabric was obtained from Fiberglast (Brookville, OH) and prepared for testing following identical methodologies as the synthesized PPTA.

*p*-Phenylenediamine (PPD) was prepared from waste PET via two reactions. First, ammonolysis was performed on PET to produce terephthalamide. Parr reactors (75 mL) were loaded with 6.0 g of waste PET, 40 mL of 7N ammonia in methanol, and pressurized using nitrogen to 40 bar. Reactors were vigorously stirred (800 rpm) at 125 °C for 48 h before being cooled and vented. The solid terephthalamide (TPD) was separated by filtration and dried in a vacuum oven overnight at 50 °C (yield 80 wt %). The second reaction was the well-known Hofmann rearrangement;^34−36^ to begin this reaction, 6.0 g of sodium hydroxide was added to 50 mL of water in a 125 mL three neck round-bottom flask placed in an ice bath. Using a syringe, 1.5 mL of liquid bromine was added to the solution to form sodium hypobromite. TPD (4.0 g) was added to the solution and mixed for 1 h. The flask was moved to an oil bath and held at 80 °C for 20 min to enable rearrangement to PPD. The mixture was filtered and PPD was extracted from the water solution with 10 washes of ethyl acetate (1:1 by volume) and concentrated by rotary evaporation. The PPD was purified via sublimation to generate a monomer of high purity (>99.5 mol %). Impure PPD was placed in a round-bottom flask with a vacuum connection. Sublimation occurred under 60 Torr and 40 °C. This process was performed until all the recovered PPD was purified, resulting in an overall yield of 64 wt % of PPD from waste PET.

Terephthalic acid (TPA) was obtained through base-catalyzed hydrolysis of PET following the procedure of Oku et al.^37,38^ Briefly, potassium hydroxide (2.25 kg), ethanol (9.56 kg), and waste PET (2.90 kg) were mixed at 45 °C for 16 h using an overhead mixer in an open stainless-steel vessel.^15,37^ The mixture formed a light tan colored slurry to which 10 L of water was added. The resulting mixture was filtered and TPA was precipitated by adding hydrochloric acid until the pH was about 2. The resulting TPA was rinsed and then dried for 24 h in a convection oven at 100 °C and pulverized before use. The yield of TPA was 98 wt %.

Terephthaloyl chloride (TCl) was obtained by chlorinating the TPA obtained from hydrolysis of PET. In a heated round-bottom flask, 20 g of TPA, 30 mL of thionyl chloride, and 5 mL of dimethylformamide (DMF) were refluxed at 80 °C for 5 h.^21,24^ The vessel was sealed and allowed to cool; excess thionyl chloride was removed through a combination of decanting and subsequent distillation. The resulting crude product was recrystallized from anhydrous diethyl ether to high purity (>99.9 mol %). The overall yield from waste PET after crystallization was 48 wt %.

To synthesize PPTA, 150 mL of dry NMP with 20 wt % dry calcium chloride was placed in a flame-dried three neck flask with an overhead mixer equipped with a glass rod and Teflon blade. The mixture was rapidly stirred under argon for 20 min. The flask was then placed in an ice bath and PPD was added at a concentration of 0.5 M. Once the temperature equilibrated, an equimolar amount of TCl was added to the flask and the reaction continued for 4 h, resulting in a viscous, gel-like product. An excess amount of water was added, and the resulting gel was mechanically broken up. The product was rinsed with methanol and water before being dried under vacuum at 60 °C to a constant weight.^17,20,22^

Monomer purity was determined by measuring melting point depression on a TA Q200 DSC (differential scanning calorimetry).^39−41^ About 7 mg of sample was added to a hermetic aluminum pan, an empty pan served as reference. The sample was heated from 10 °C below to 5 °C above the literature value of the melting point^42^ at a rate of 0.5 °C/min. Purity was determined using the van’t Hoff equation.

As is usually practiced, the PPTA product was analyzed using viscometry to indicate molecular weight in accordance with the literature.^18,19,28,43−45^ The inherent viscosity was measured using a 1C Cannon Ubbelohde Viscometer at a concentration of 0.5 g/dL polymer dissolved in 98% sulfuric acid at 30 °C. The inherent viscosity is defined by eq 1,
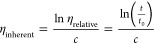
1where *t* is the solution drain time, *t*_0_ the solvent drain time, and *c* is concentration in g/dL.

Nuclear magnetic resonance (NMR) spectroscopy was conducted using solution and in the solid state. Liquid solutions of TPA,TPD, and PPD in deuterated dimethyl sulfoxide (DMSO) were created and compared with standards of the compounds using ^1^H NMR. Deuterated chloroform was used as the solvent for TCl. Samples were ran on a Bruker 500 MHz with 2 s relaxation delay and 32 scans. PPTA was analyzed using solid-state NMR using a Varian InfinityPlus 400 MHz spectrometer equipped with a 6 mm CPMAS probe. The contact time was 4 ms, the acquisition time was 10.2 ms, and the spectral width was 50.45 kHz. Carbon NMR scans were taken using a frequency of 100.52 MHz. All NMR data were analyzed on Mnova NMR software.

Attenuated total reflection (ATR) spectra were taken of solid-state samples using a Nicolet is50R spectrometer equipped with a DTGS detector using 32 scans. Samples were predried in a convection oven at 100 °C to remove moisture.

Thermogravimetric analysis (TGA) was performed on a TA Instruments Q500. The sample pan treated with a propane torch to remove residues and tared; about 15 mg of sample was used. After equilibration at 50 °C, the sample was heated at a rate of 10 °C/min to 600 °C.

Optical microscopy was conducted on PPTA in sulfuric acid to determine the onset of a liquid crystalline phase, which forms due to the rigid-rod structure of PPTA.^46−48^ A concentrated solution was prepared by heating in sulfuric acid and then diluted to concentrations between 5 and 30 wt %. Samples were smearing onto a microscope slide and a coverslip added and imaged under both linear and cross-polarization.

## Results and Discussion

**Figure 3:**
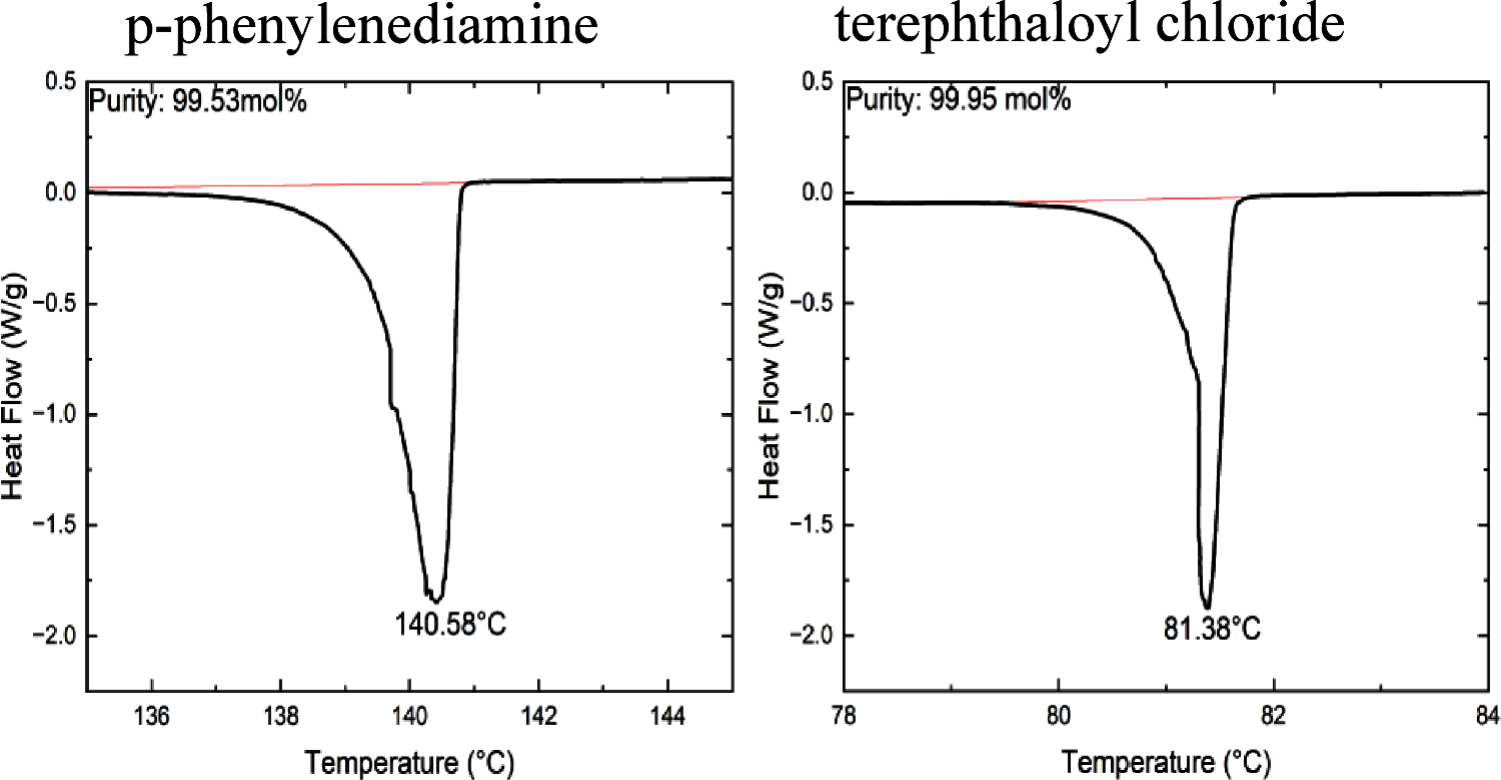
DSC traces. Monomer purities were determined using DSC for PPD (left) and TCl (right) created from waste PET.

PPTA is commercially synthesized by step-growth polymerization of PPD and TCl,^20^ and these monomers were successfully obtained from waste PET thermoform containers. PET was hydrolyzed and converted to high purity TCl (>99.9 mol % by melting point) with an overall yield of 48 wt %. The ammonolysis of PET, subsequent Hofmann rearrangement, recrystallization, and sublimation produced high purity PPD (>99.5 mol % by melting point) with an overall yield of 64 wt %. The starting material and monomer products are shown in Figure 2 and the DSC traces used to determine purity are shown in Figure 3 (see also the Supporting Information).

**Figure 4:**
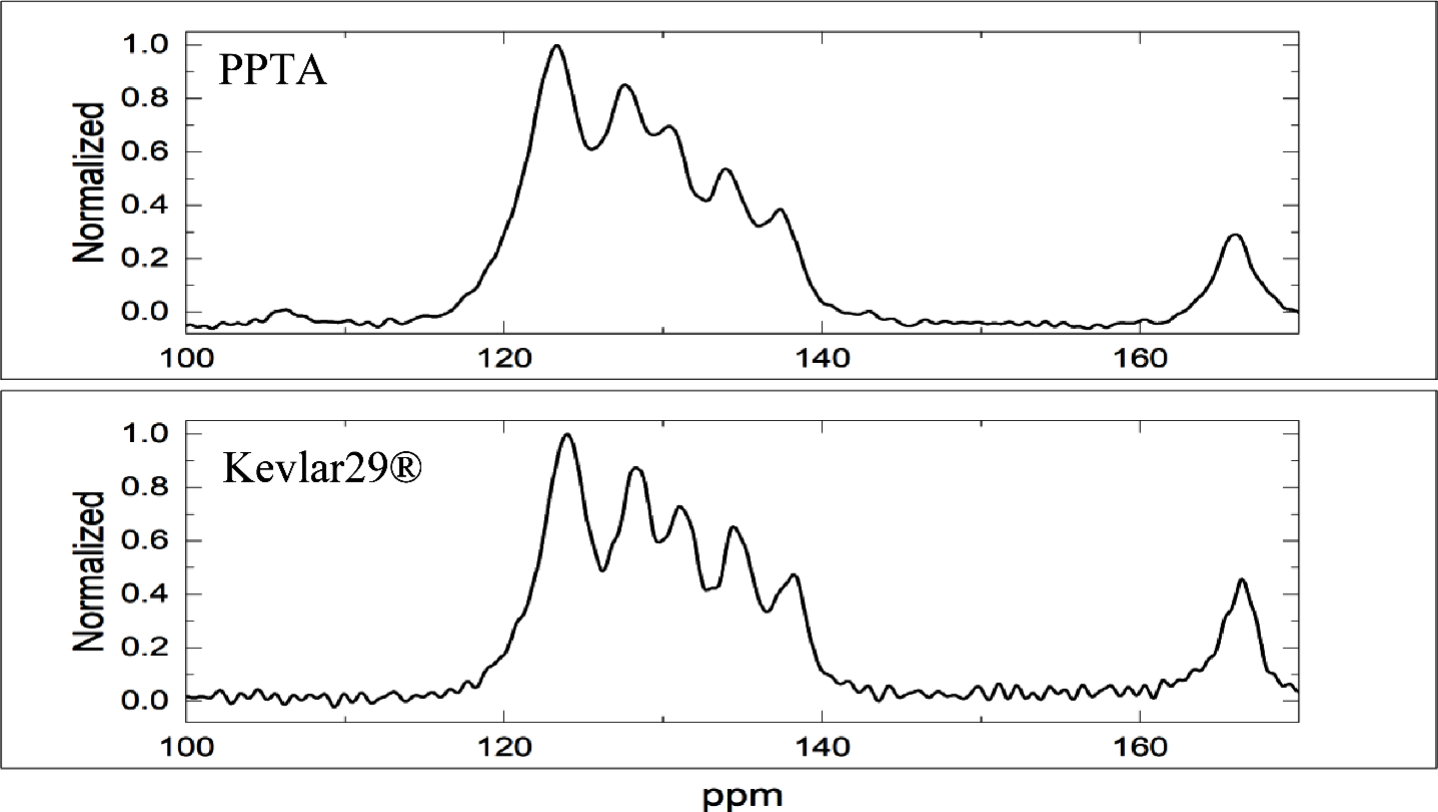
Solid state NMR. PPTA synthesized from waste PET is shown on top and the commercial Kevlar29 fabric on the bottom. The same peaks are observed in the standard and successfully synthesized PPTA.

Solid state 13C NMR conclusively demonstrates PPTA was successfully synthesized from the waste-derived monomers. Figure 4 shows the spectrum for a synthesized sample along with the spectrum for a commercial Kevlar 29 sample. The same polymer backbone structure is observed in the two spectra. There are five electronically unique carbons present in the repeat unit of PPTA, one in the aramide linkages and four in the aromatic rings. The peak at 165 ppm can be assigned to the carbonyl carbon of the amide group.^49^ The remaining unique carbons are attributed to the multiplet within the aromatic region. Importantly, the same peaks are observed in the PPTA obtained from waste PET and the sample of commercial Kevlar 29.

**Figure 5:**
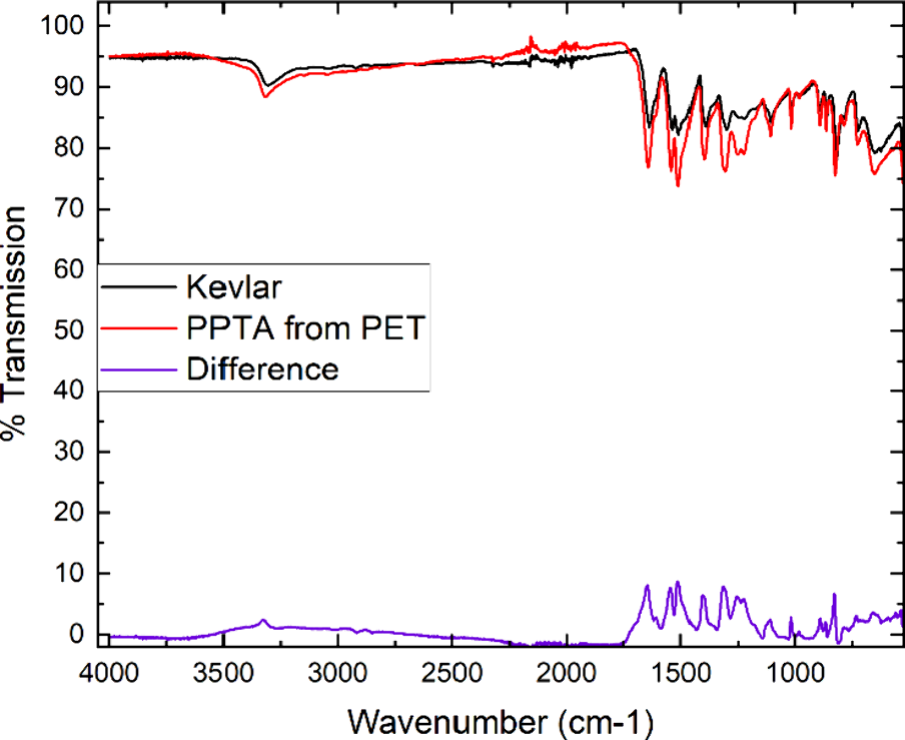
ATR spectra. No significant variations in peaks are evident between the two samples. The difference when the two peaks were subtracted from each other is close to zero.

ATR is also used to compare the PPTA synthesized from waste PET to the Kevlar 29 standard. Spectra are presented in Figure 5. As with solid state NMR, no significant variations in the peaks were observed between the synthesized PPTA and the commercial Kevlar29. The matching spectra indicates identical functional groups are present in each material. Specifically, an amide peak, characterized by a broad absorbance range, is present at 3310 cm^–1^. Samples show evidence of acid end groups around 3000 cm^–1^ and the corresponding carbonyl group at 1680 cm^–1^. All the observed peaks are consistent with the desired product. When the two spectra were subtracted, the resulting graph remains close to zero, further emphasizing the similarities of the two samples.

Another property of PPTA is its ability to form lyotropic liquid crystalline phases. PPTA has very little rotational freedom around backbone bonds due to its aromatic rings and planar amide linkages; it has a “rigid-rod” structure. Above a critical concentration, chains align to create a nematic liquid crystal solution.^46,50,51^ Literature reports that at around 18 wt % in 98% sulfuric acid at 30 °C is where typical PPTA creates a liquid crystalline phase.^52,53^ A liquid crystal phase is observed with the synthesized PPTA at 20 wt % (see Supporting Information). The polymer rich phase has a light tan color and is extremely gel-like but exhibits hallmark birefringence when observed under cross-polarization. The polymer-deficient phase is dark brown and less viscous than the gel-like phase.

Viscometry is the most common method for measuring the molecular weight of PPTA. The commercial Kevlar29 fabric yielded an inherent viscosity of 5.2 dL/g while the inherent viscosity achieved using upcycled PET was 4.8 dL/g.

**Figure 6:**
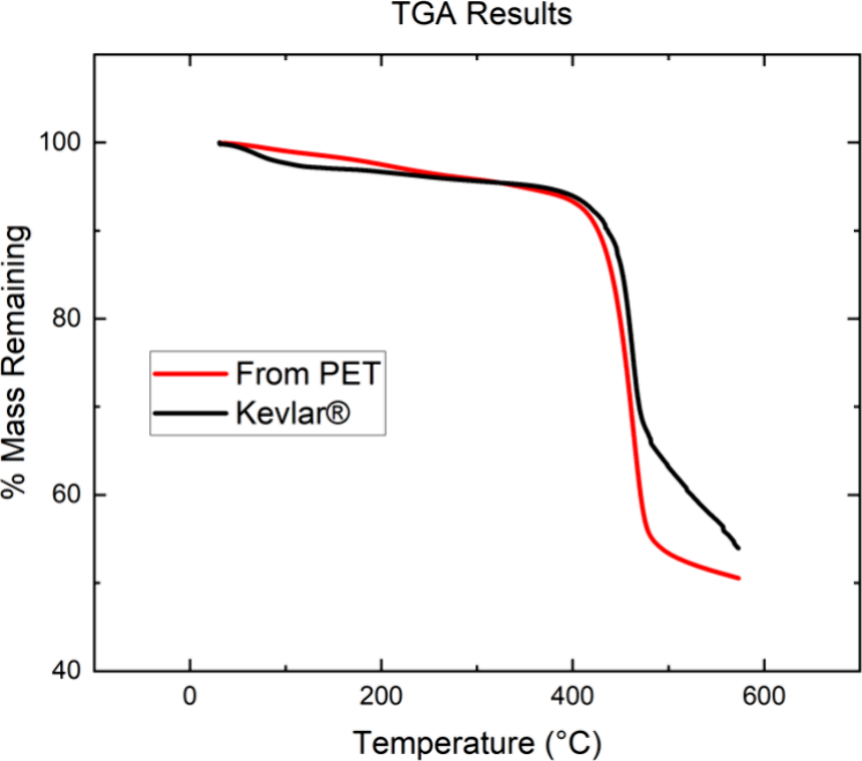
Comparative thermal properties. The onset temperatures for decomposition are very similar; 430.5 °C for the PPTA and 431.6 °C for Kevlar29.

Because heat resistance is an important property of polyaramides, TGA was used to compare the waste-sourced PPTA to the Kevlar29 standard. The results of these tests are shown in Figure 6. The measured onset temperature for the synthesized PPTA is 430.5 °C which compares favorably with the value of 431.6 °C measured for the standard. The matching onset temperatures indicate essentially identical thermal stability. The corresponding thermal behavior further confirms that high-valued PPTA was obtained from zero-valued waste PET.

## Conclusions

For the first time, high-value PPTA has been synthesized using 100% recycled carbon atoms derived from zero-valued PET waste. This new and economically feasible upcycling route is accomplished through the innovative and novel combination of known reactions. PPD is obtained by ammonolysis of PET followed by the Hofmann rearrangement. TPA is obtained through hydrolysis of PET, and TCl is synthesized through chlorination. The PPTA obtained is comparable to commercial Kevlar29 when investigated using solid-state NMR, ATR, and TGA. Additionally, the polymeric nature is confirmed through viscometry and observation of a nematic liquid crystalline phase. The presented set of reactions provides a new viable means for deriving value-added monomers and polymers from presently unused PET thermoform waste streams.

Given the 20-fold increase in value between reconstituted PET and PPTA, the present approach represents a bona fide exercise in polymer upcycling. Starting from effectively zero-valued waste PET thermoforms, the embedded carbon was recovered and successfully upcycled into high-value PPTA, best known by its trade name Kevlar. This lifesaving material is widely used by members of the military, police, and other first responders; the present study shows the application of the principles of Green Chemistry to go from zero to hero! Future work on this subject includes a complete TEA/LCA and an ASPEN process flow model to establish the detailed economic viability of producing the PPD monomer from waste PET.

## Data Availability

The data supporting this article have been included as part of the Supporting Information.

## Supporting Information Available

Data and photographs relating to monomer syntheses and purification; differential scanning calorimetry, LCMS, and NMR spectra for monomer analysis; and viscometry data and cross-polarized observation of the PPTA liquid crystalline phase (PDF)
