# Supplementary material for: From Zero to Hero: Polymer Upcycling through Transformation of Waste PET Thermoforms into Kevlar
Source: ACS Appl Polym Mater. 2025 Apr 24;7(9):5475–81. doi: 10.1021/acsapm.5c00191 (PMC12070366; doi:10.1021/acsapm.5c00191)
Supplement: Supplementary file 1 — ap5c00191_si_001.pdf [file ap5c00191_si_001.pdf]

# Supplemental Information for From Zero to Hero: Polymer upcycling through transformation of waste PET thermoforms into Kevlar®

*Elanna P. Neppel, Richard-Joseph L. Peterson , Lars Peereboom , and John R. Dorgan**

AUTHORS ADDRESS: Department of Chemical Engineering and Materials Science,
Michigan State University, East Lansing, MI 48824 USA

The supporting information includes data relating to monomer synthesis and purification as well as the polymer characterization. The Parr reactor system (Fig. S1) was used to create terephthalamide from PET using ammonolysis. The set up for the Hofmann reaction (Fig. S2-S3) was used to synthesize para-phenylene diamine (PPD). Differential scanning calorimetry (Fig.S4-S7) was used to determine monomer purity for both PPD and terephthaloyl chloride (TCl). LCMS (Fig S8-S9) shows impurities in the PPD before and after sublimation. NMR (Fig. S10-S13) was used to compare the monomers obtained to standards purchased from Sigma Aldrich. Viscometry was conducted on PPTA obtained for comparison with literature values and a commercial sample. Microscopy (Fig. S14-S15) confirmed the presence of a liquid crystalline phase.

**Email address: jd@msu.edu*

# Table of Contents

**3.0 Supplemental materials** ........ S3
*3.1 Parr multi-reactor system* ........ S3
*3.2 Hofmann reaction setup* ........ S3
*3.3 Differential scanning calorimetry (DSC)* ........ S5
*3.4 LCMS data* ........ S9
*3.5 NMR data* ........ S11
3.6 Viscometry ........ S14
3.7 Microscopy ........ S15

## 3.0 Supplemental materials

### *3.1 Parr multi-reactor system*

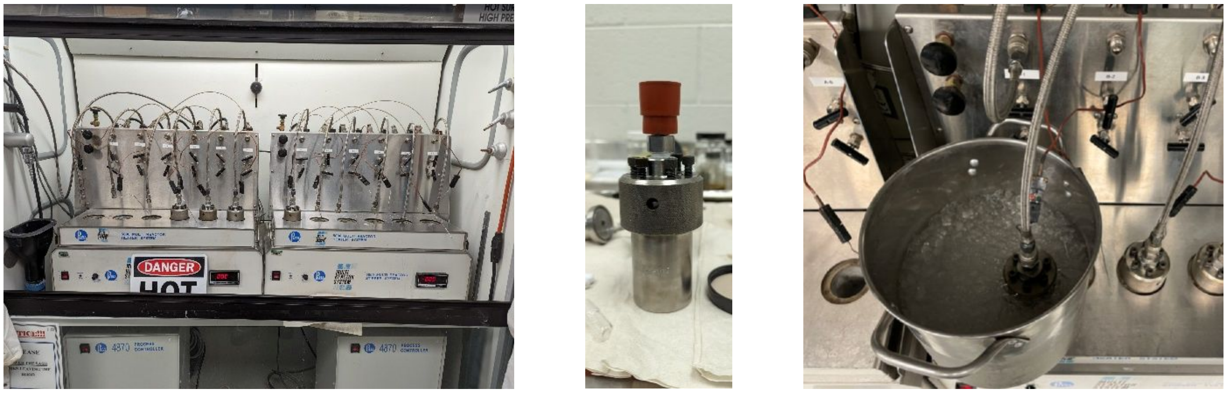


(a) (b) (c)

Figure S1. The Parr reactor system used in the ammonolysis of PET described in this study: (a) The multi-reactor system can hold 6 independent reactors connected to a gas manifold, (b) one reactor fitted with rubber septum for loading reactants, and (c) quenching the reaction by submersing a reactor in an ice bath.

### *3.2 Hofmann reaction setup*

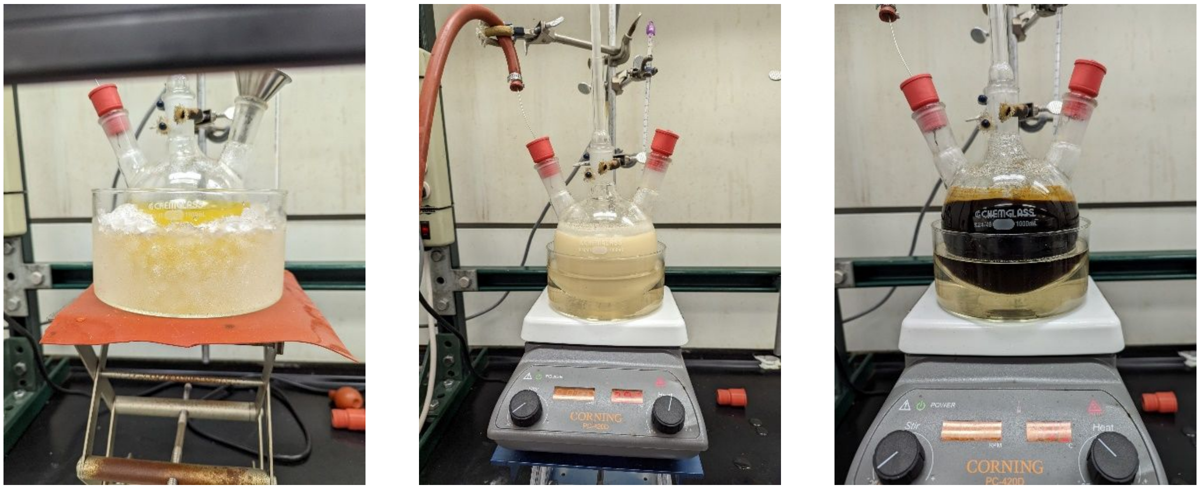


Figure S2. The Hoffman Rearrangement on TPD derived from waste PET. The cold sodium hypobromite solution (left) is cooled in an ice bath. Then TPD is mixed in (center) to the solution and mixed for one hour. The flask is then moved to a hot oil bath at $80^{0}$C (right) and a color change occurs. The halogenation happens while the mixture is in the ice bath and the rearrangement is accomplished by heating to $80^{0}$C.

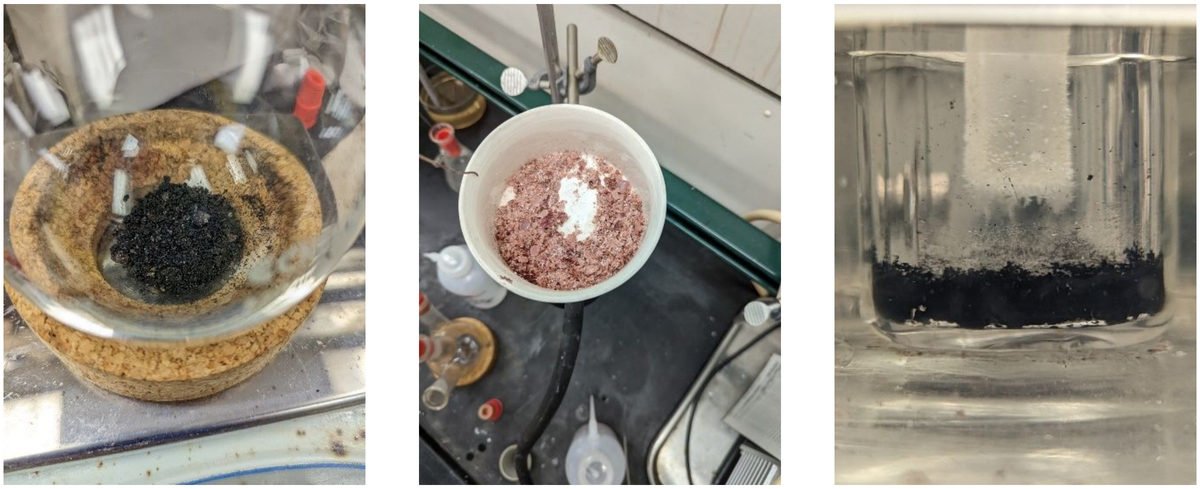

Figure S3. PPD purification. Crude PPD from waste PET (left), recrystallized from ethanol (center), and high-purity crystal from sublimation(right). Purities determined by melting point depression are  88,  98.2 and 99.4 mol%, respectively.

## 3.3 Differential scanning calorimetry (DSC)

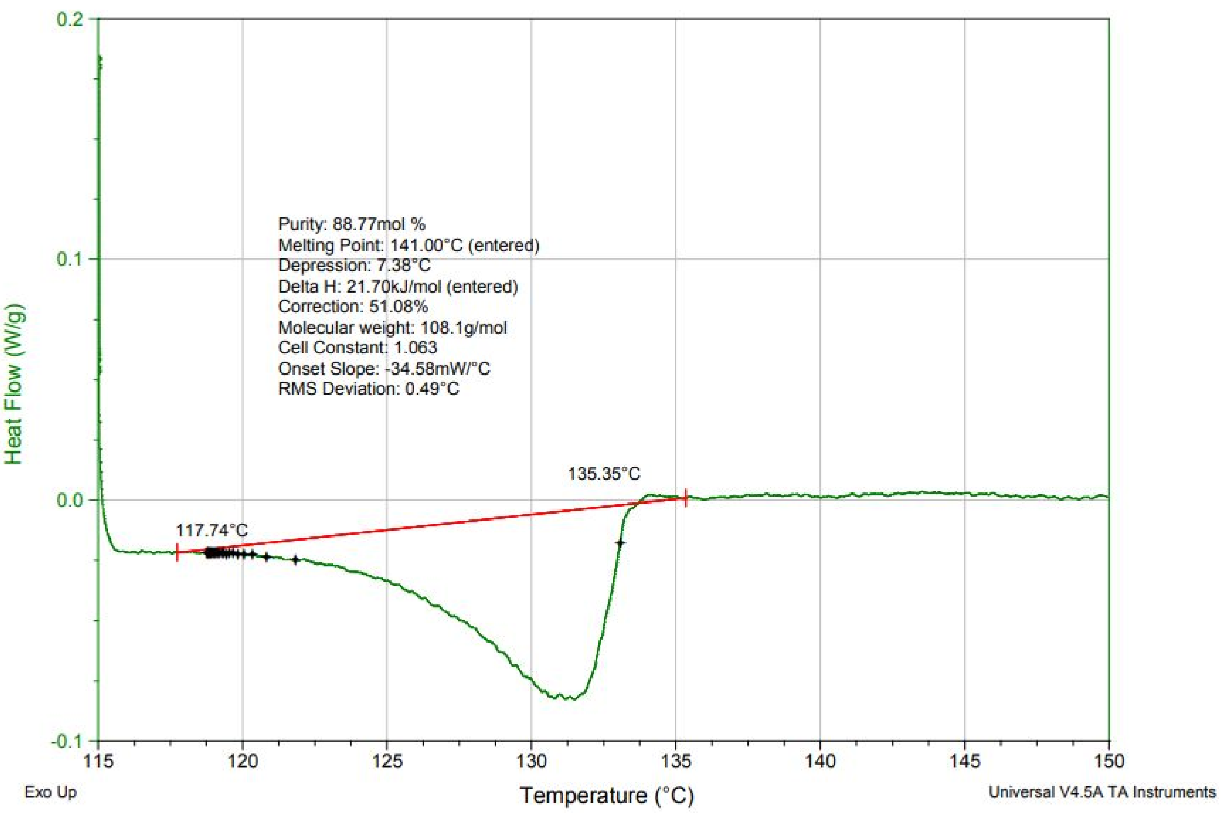


Figure S4. DSC trace of crude PPD produced from waste PET.

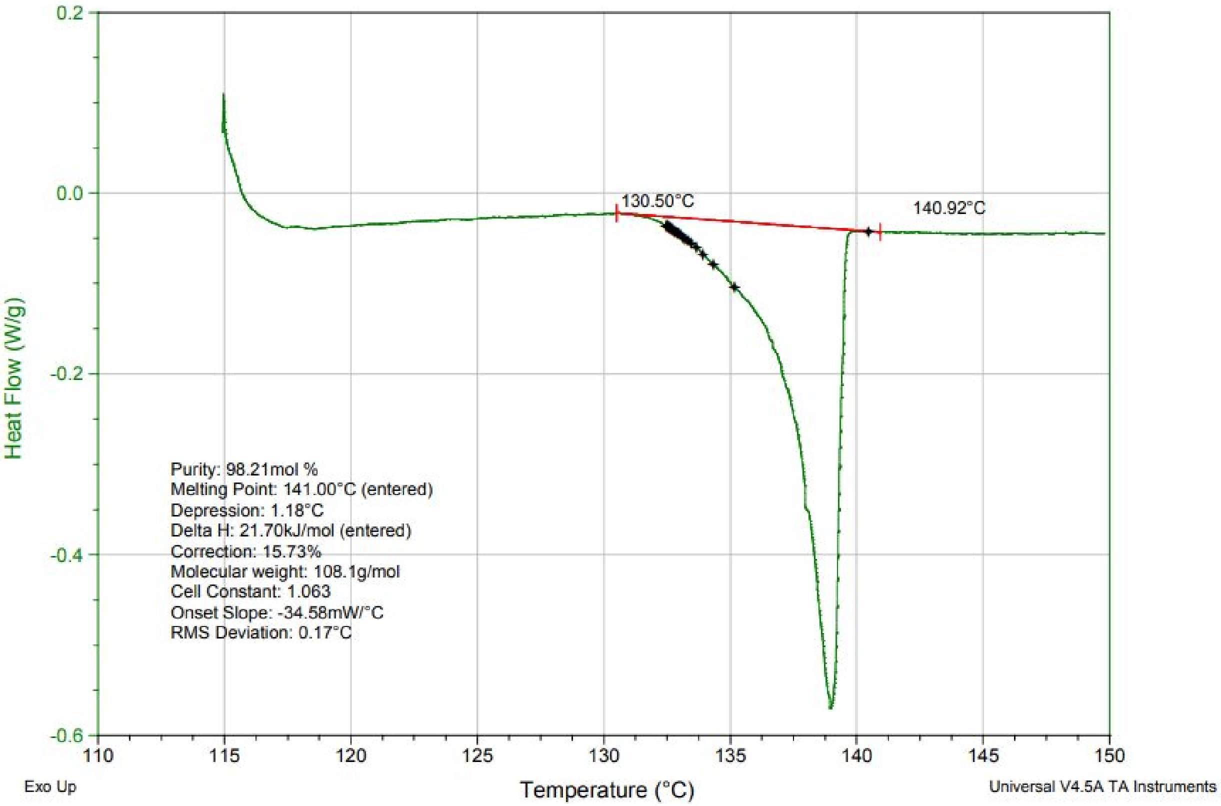


Figure S5. DSC trace of PPD produced from waste PET after recrystallization from ethanol.

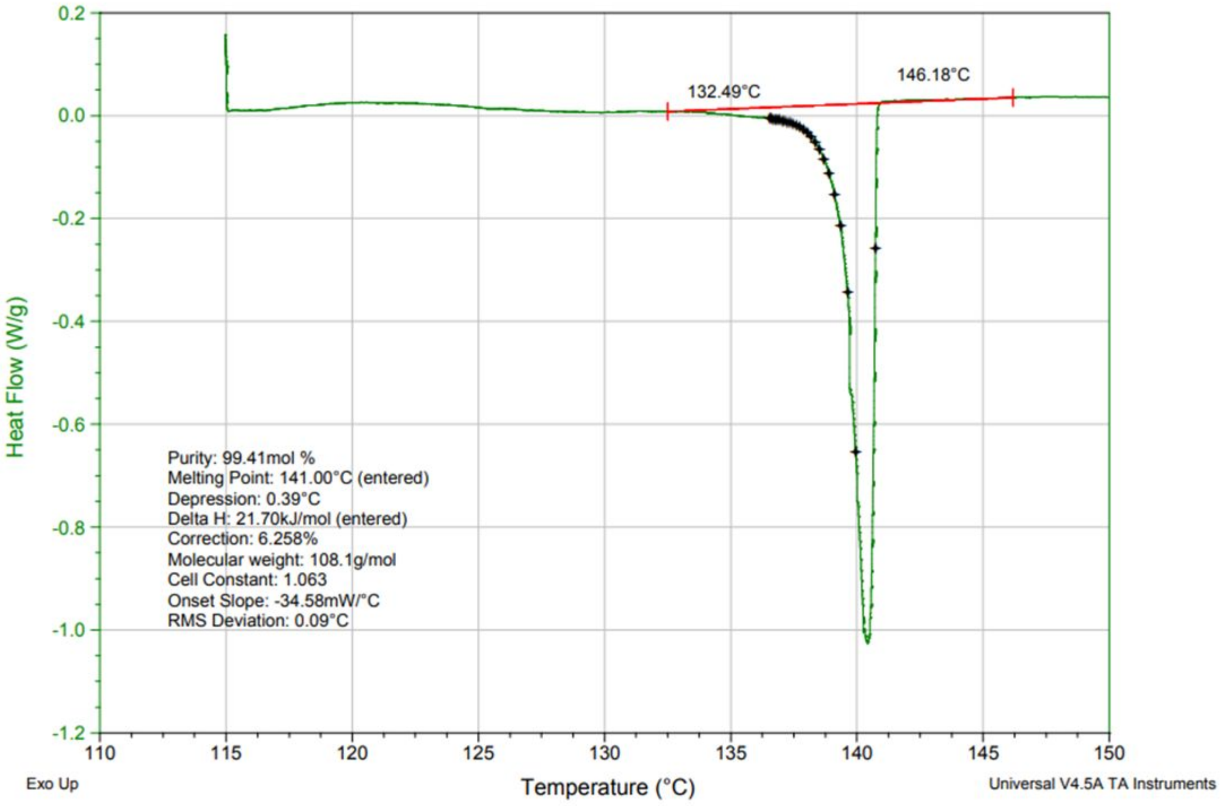


Figure S6. DSC trace of PPD produced from waste PET after sublimation.

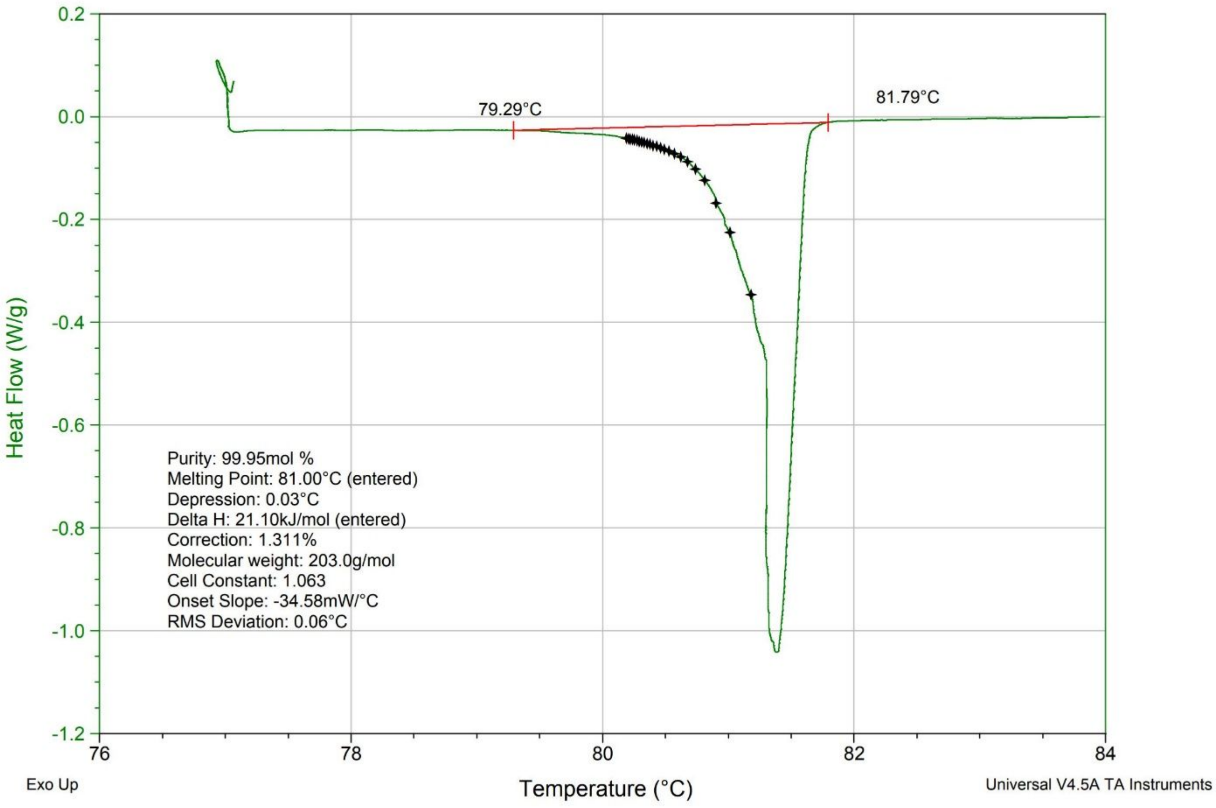


Figure S7. DSC trace of TCl produced from waste PET after a recrystallization from diethyl ether.

## 3.4 LCMS data

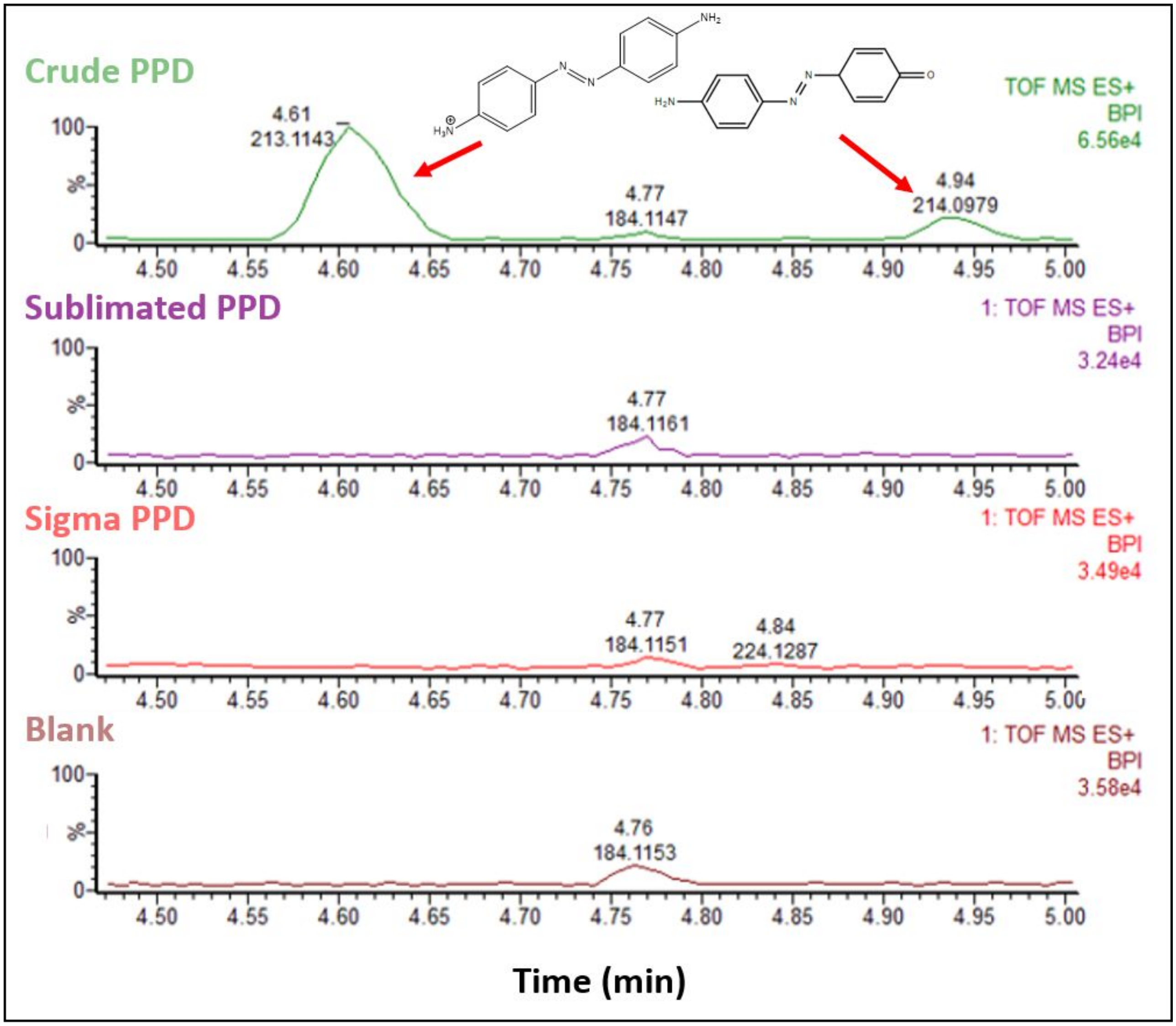


Figure S8. LCMS data for PPD at various purification stages. Lowest chromatogram is the column blank, above it is the material purchased from Sigma ("Sigma PPD"). The topmost chromatogram is the crude PPD showing contaminants having masses of 213.1 and 214.1 and their likely molecular structures; second from top is the sublimated material.

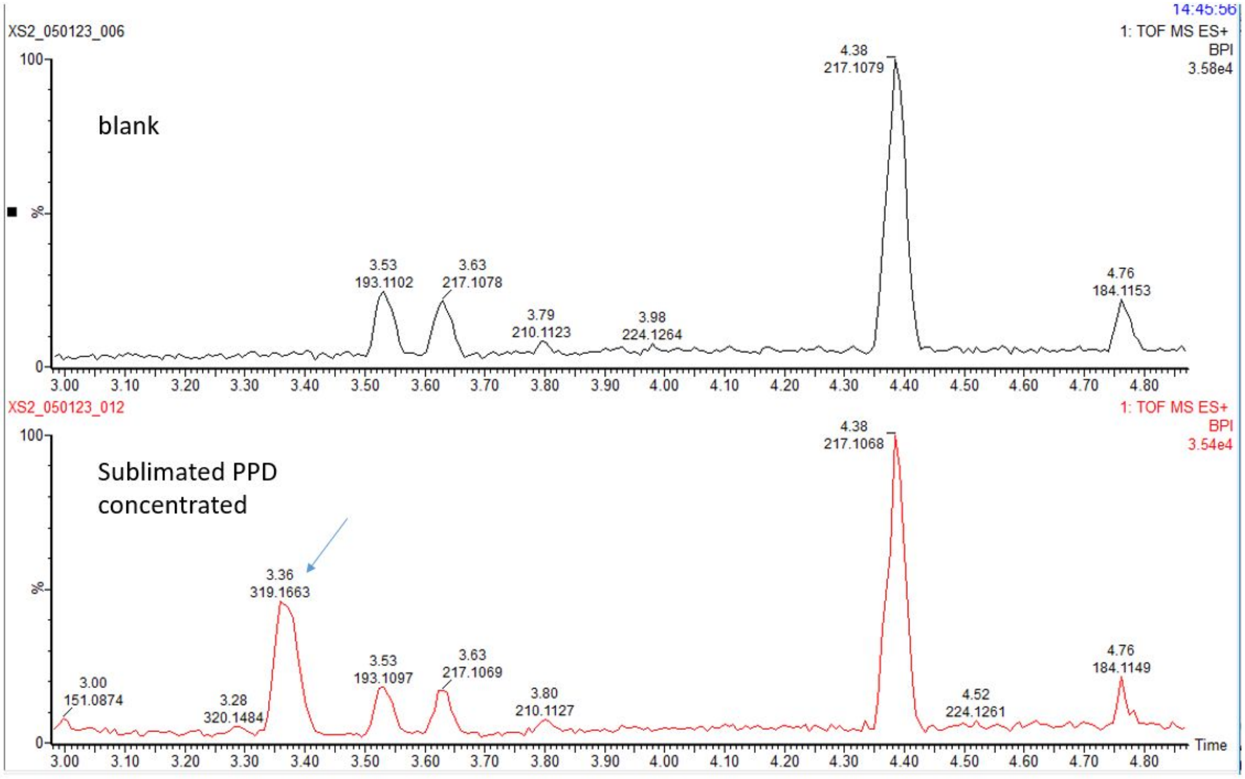


Figure S9. LCMS data for the sublimated PPD after oxidation. After 24 hours Brandowski's base is clearly present, having an exact molar mass of 319.1668 Da.

## 3.5 NMR data

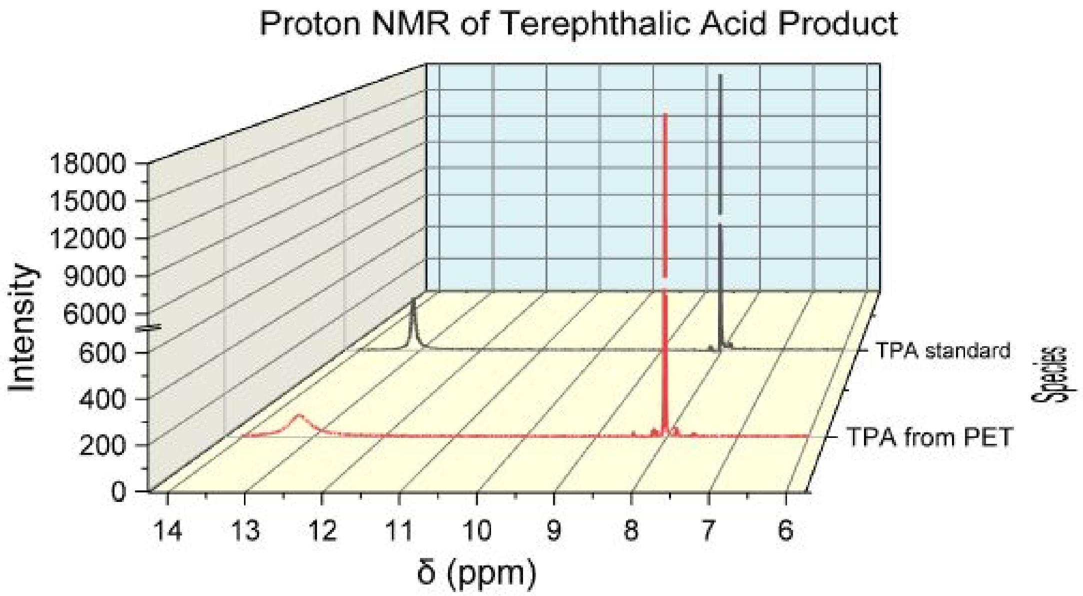


Figure S10. Proton NMR (500MHz) spectra of a terephthalic acid standard and terephthalic acid from waste PET. Terephthalic acid $^{1}H$ NMR ((500MHz, DMSO) δ 13.20 (s, 2H), 8.03 (s, 4H)). Peak broadening at 13.20 ppm is attributed to residual water.

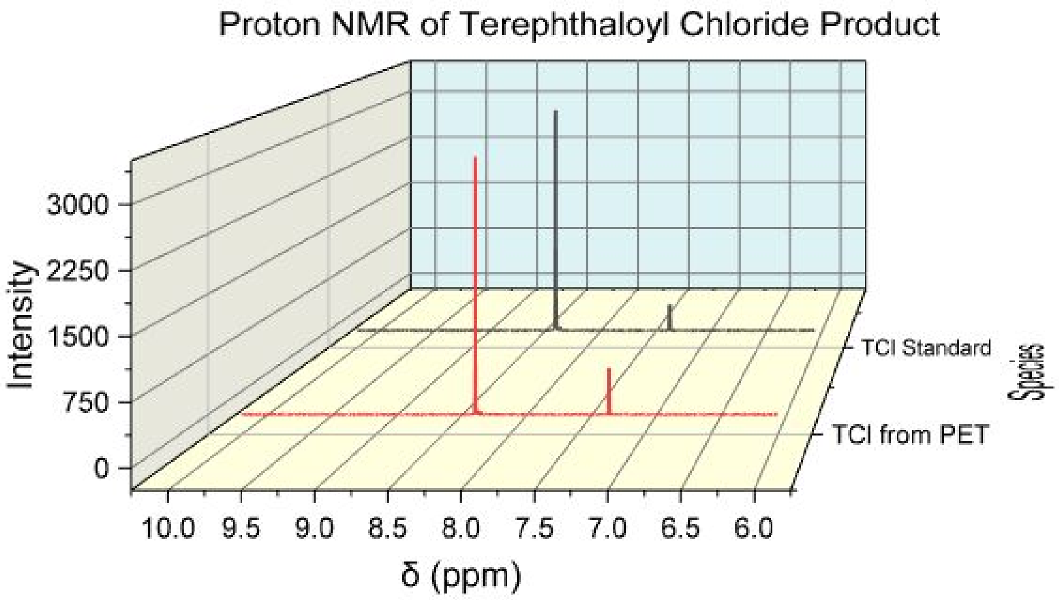


Figure S11. Proton NMR of a terephthaloyl chloride standard and terephthaloyl chloride from waste PET. Terephthaloyl chloride $^{1}H$ NMR ((500MHz, CDCl3) δ 8.27 (s, 4H)).

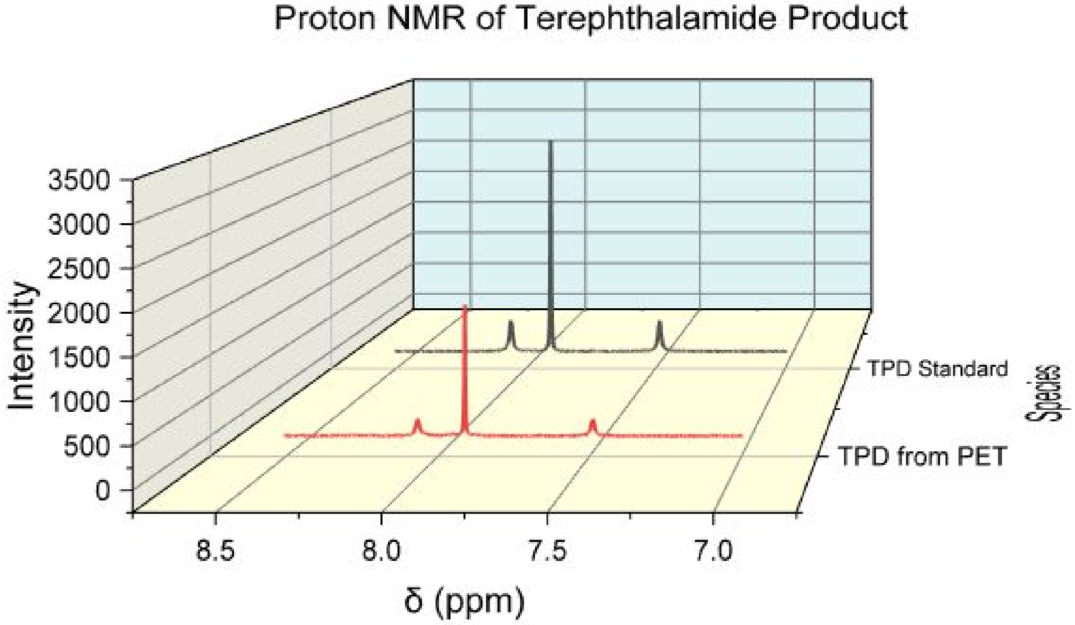


Figure S12. The proton NMR of a terephthalamide standard and terephthalamide from waste PET. Terephthalamide $^{1}H$ NMR ((500MHz, DMSO) δ 8.06 (s, 2H), 7.91 (s, 4H), 7.49 (s, 2H)).

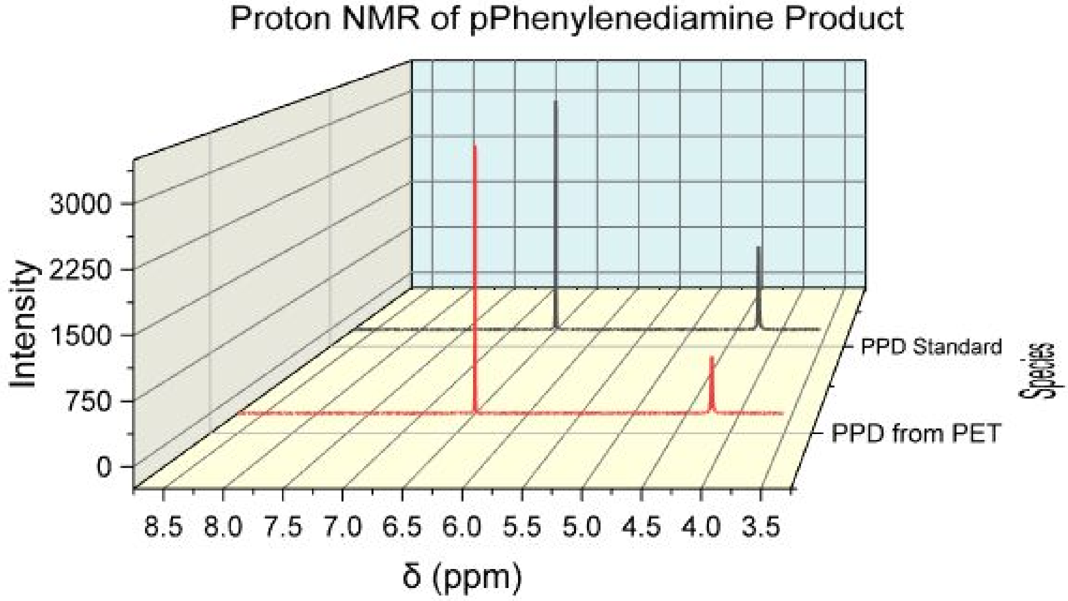


Figure S13. The proton NMR of a p-Phenylenediamine standard and p-Phenylenediamine from waste PET. P-phenylenediamine $^{1}$H NMR ((500MHz, DMSO) δ 6.33 (s, 4H), 4.16 (s, 4H)).

## 3.6 Viscometry

| | Pure 98% Sulfuric acid | PPTA from PET Solution (0.5 g/dL) |
|---|---|---|
| Time (minutes:seconds) | 4:39.31 | 11:06.12 |
| | 4:38.21 | 11:07.48 |
| | 4:38.12 | 11:08.41 |
| | 4:39.27 | 11:07.38 |
| | 4:39.51 | 11:06.48 |
| Average | 4:38.88 | 11:07.17 |

Inherent viscosity is used as a measurement of the molecular weight of PPTA. To determine the inherent viscosity, S1 is used.

$$\eta_{inh} = \frac{\ln\left(\frac{t}{t_0}\right)}{c} \quad \textit{(S1)}$$

Where $\eta_{inh}$ is the inherent viscosity, t is the time in seconds the solution takes to pass through the viscometer capillary, $t_0$ is the time in seconds the solute free solution takes to pass through the viscometer capillary, and c is the concentration of the solution in g/dL.

## 3.7 Microscopy

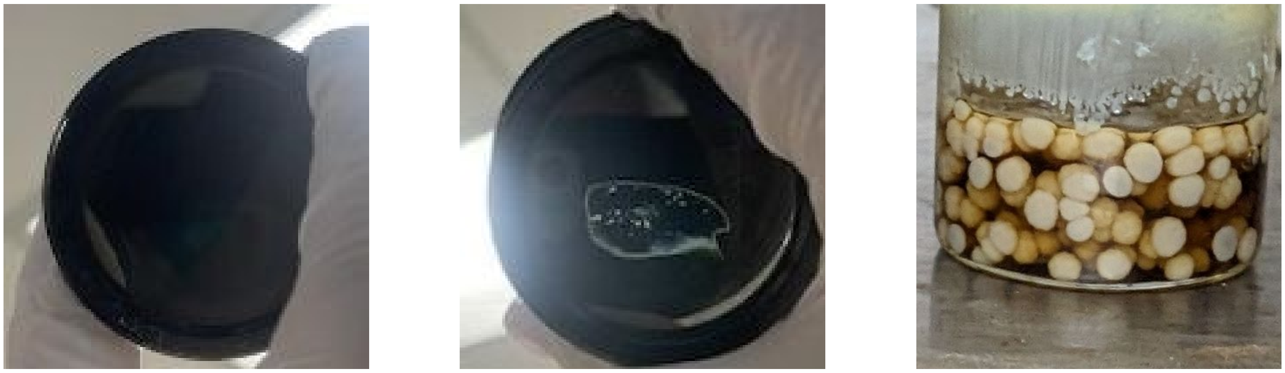

Figure S14. Cross polarizers arranged at 90 with no sample in between the two (left) and with a 10wt% PPTA synthesized from waste PET dissolved in 98% sulfuric acid (center). The cross polarizers were in the same orientation for both images. The birefringence is indicative of liquid crystalline behavior. At room temperature, two phases were observed (right). The more gel-like, polymer rich phase (light tan) and a more liquid-like phase (dark brown). Such phase separation is expected for liquid crystalline polymers of high molecular weight.

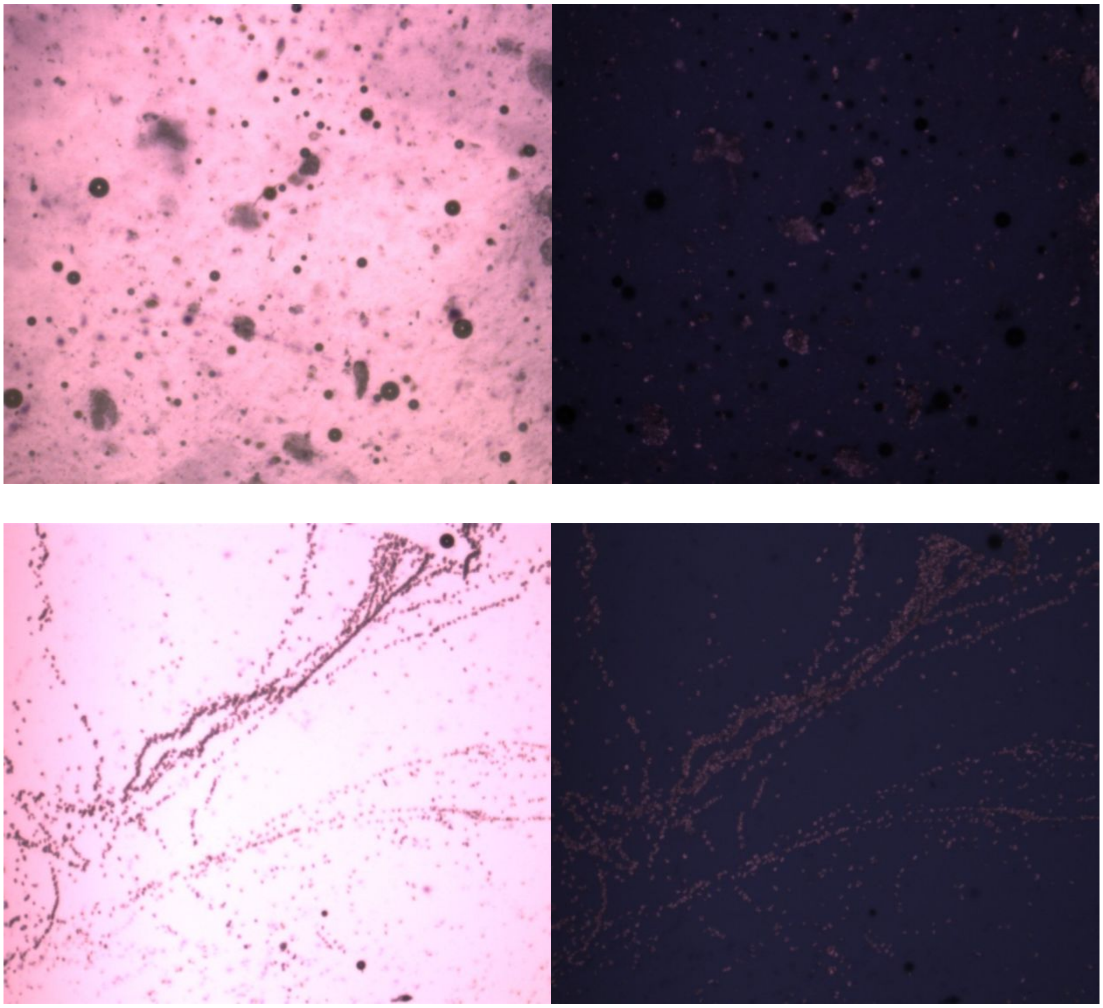

Figure S15. Standard Kevlar29 under a microscope without cross-polarizers (top left) and under cross polarizers (top right) compared to lab synthesized PPTA from waste PET without cross polarizers (bottom left) and under cross polarizers (bottom right). In both samples, light was able to pass through most areas in the sample without cross-polarizers with ease. The darker areas turned into the lighter parts when cross-polarization was added (right). This is indicative of sections having a liquid crystalline region, as light was able to pass through regions it was unable to without it. Both samples exhibit this property.
